# Case Report: Monoclonal gammopathy associated enteropathy: a potential MGCS related phenotype with response to plasma cell directed therapy

**DOI:** 10.3389/fmed.2026.1881584

**Published:** 2026-09-03

**Authors:** Guo Chen, Li He, Yuan Yuan Chen, Peng Yang, Jing Tan

**Affiliations:** 1 Department of Hematology, The Third People’s Hospital of Chengdu, Chengdu, Sichuan, China; 2 Department of Pathology, The Third People’s Hospital of Chengdu, Chengdu, Sichuan, China

**Keywords:** chronic diarrhea, inflammatory enteropathy, monoclonal gammaglobulinemia, monoclonal gammopathy of clinical significance (MGCS), plasma cell disorder

## Abstract

**Background:**

Chronic inflammatory diarrhea with preserved mucosal architecture remains diagnostically challenging when inflammatory bowel disease, autoimmune enteropathy, and infection are excluded. The potential contribution of monoclonal gammopathy in such cases remains poorly defined.

**Case presentation:**

We report two patients with long-standing, treatment-refractory chronic diarrhea who underwent comprehensive clinical, histopathologic, and hematologic evaluation. Both demonstrated a consistent intestinal pattern characterized by preserved mucosal architecture, absence of crypt distortion or epithelial apoptosis, diffuse polyclonal plasma cell–rich inflammation, and negative Congo red staining. Each patient was found to have a circulating monoclonal immunoglobulin. Conventional therapies were ineffective. Initiation of plasma cell–directed therapy resulted in rapid and sustained resolution of diarrhea, accompanied by marked reduction or disappearance of the monoclonal immunoglobulin.

**Conclusion:**

These findings suggest that circulating monoclonal immunoglobulins may contribute to a previously underrecognized form of inflammatory enteropathy within the spectrum of monoclonal gammopathy–associated disorders. Further studies are required to determine whether this represents a distinct clinical entity.

## Introduction

Chronic inflammatory diarrhea with preserved mucosal architecture remains a diagnostic challenge when established etiologies such as inflammatory bowel disease, autoimmune enteropathy, and infection are excluded. In this setting, the potential contribution of systemic or hematologic factors is often underrecognized.

Monoclonal gammopathies of clinical significance (MGCS) comprise a heterogeneous group of disorders in which a B-cell or plasma-cell clone produces a biologically active monoclonal immunoglobulin that leads to end-organ dysfunction (1). While renal, neurologic, and dermatologic manifestations of MGCS are well recognized, these disorders illustrate a broader principle: circulating monoclonal immunoglobulins can act as pathogenic mediators of organ injury even in the absence of overt tissue deposition or clonal infiltration (2). However, gastrointestinal involvement within this spectrum remains poorly characterized. In particular, whether monoclonal immunoglobulins may contribute to otherwise unexplained inflammatory enteropathy remains unclear.

Here, we describe two patients with long-standing, treatment-refractory chronic diarrhea who exhibited a similar clinicopathologic phenotype associated with monoclonal gammopathy. The clinical response to plasma cell directed therapy suggests a potential association between monoclonal gammopathy and this enteric phenotype, highlighting the need for further investigation of the role of monoclonal immunoglobulins in inflammatory enteropathy.

## Case description

### Case 1

A 63-years-old man presented with a 3-years history of chronic nonbloody watery diarrhea, progressively worsening to 4–8 bowel movements daily and accompanied by 5-kg unintentional weight loss. He appeared cachectic (body mass index, 16.4 kg/m^2^). Laboratory evaluation showed normocytic anemia (hemoglobin, 98 g/L), hypoalbuminemia (29.6 g/L), elevated inflammatory markers (erythrocyte sedimentation rate, 120 mm/h; C-reactive protein, 13.4 mg/L), and marked hyperamylasemia (557 IU/L) and hyperlipasemia (2042.5 IU/L) without clinical or radiographic evidence of acute pancreatitis.

Extensive gastrointestinal evaluation failed to identify a defined etiology. Colonoscopy showed right-sided colitis with dense lymphoplasmacytic infiltration without crypt distortion. Small-bowel endoscopy demonstrated multiple ulcers, mucosal atrophy, and stenosis. Small-intestinal biopsies showed preserved villous architecture without villous blunting or increased intraepithelial lymphocytes, with diffuse lymphocytic expansion of the lamina propria and preserved goblet and Paneth cells. Immunophenotyping revealed mixed CD20^+^ B cells and CD3^+^ T cells with mildly increased CD38^+^/CD138^+^ plasma cells. Light-chain staining demonstrated balanced κ and λ expression without IgG4 enrichment. Congo red staining was negative. IGH rearrangement was detected, with negative TCRγ and IGK rearrangements.

Serum protein electrophoresis identified an IgG-κ monoclonal protein (8.9 g/L). Bone marrow examination showed 2% morphologically mature plasma cells without light-chain restriction, not meeting criteria for multiple myeloma. Empirical therapies including antibiotics, probiotics, dietary elimination (gluten-free and dairy-free diets), and prednisone (30 mg/day for 2 weeks) failed to improve symptoms.

**FIGURE 1:**
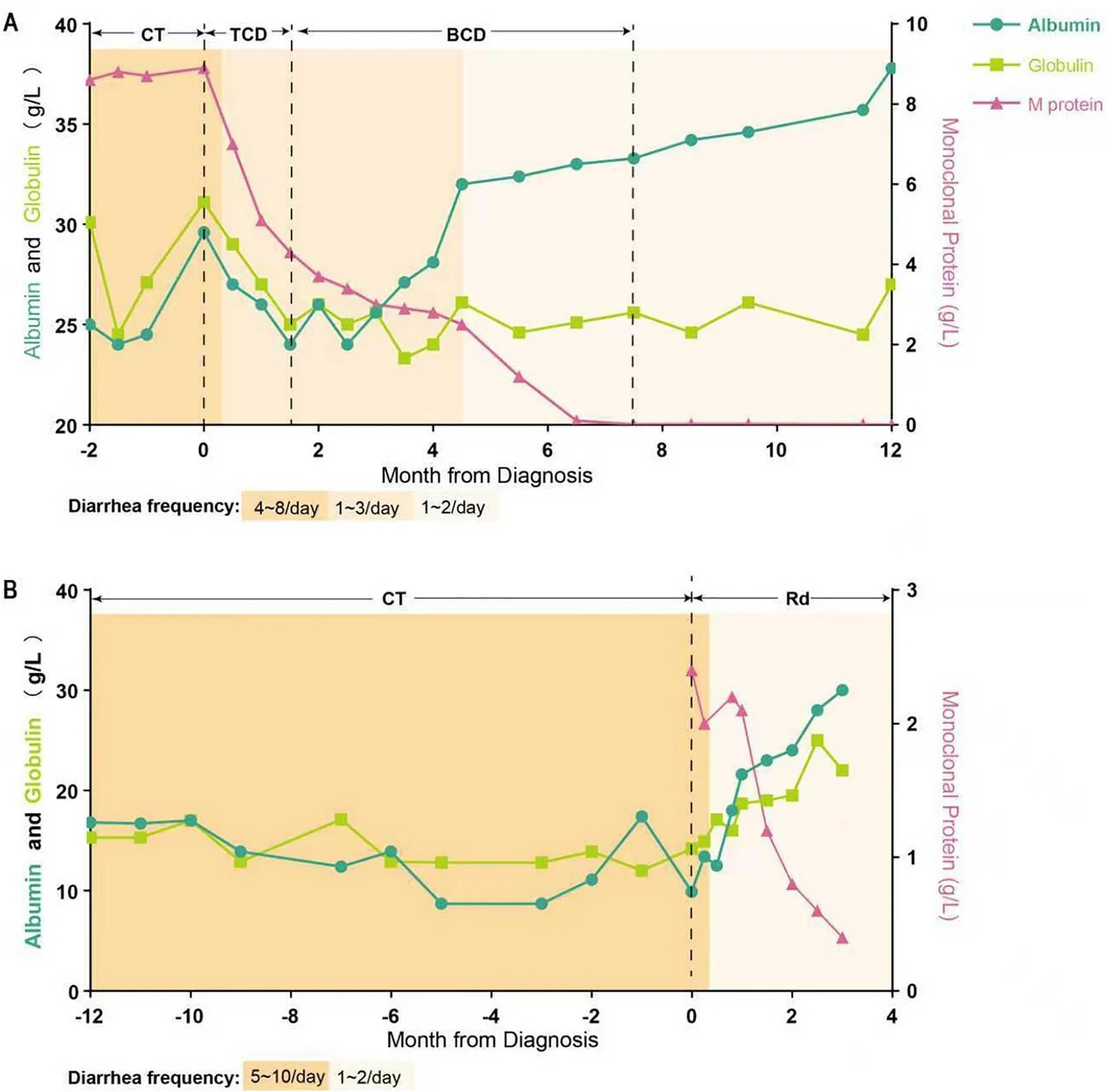
Changes in diarrhea symptoms and monoclonal proteins in two patients before and after plasma cell-targeted therapy. **(A,B)** Show that in Case 1 and Case 2, respectively, after plasma cell–directed therapy, the frequency of diarrhea decreased significantly, albumin levels increased noticeably, and monoclonal protein decreased markedly. CT, conventional therapies, including antibiotics, probiotics, gluten-free diet, and prednisone; TCD, thalidomide, cyclophosphamide and dexamethasone; BCD, bortezomib, cyclophosphamide, and dexamethasone; Rd, lenalidomide and dexamethasone.

Given the absence of an alternative etiology and the presence of monoclonal gammopathy, plasma cell–directed therapy was initiated with thalidomide (100 mg nightly), cyclophosphamide (400 mg weekly), and dexamethasone (15 mg every other day), leading to rapid symptomatic improvement (1–2 bowel movements daily). After 45 days, the monoclonal protein decreased to 4.3 g/L. Treatment was subsequently switched to a bortezomib-based regimen combined with cyclophosphamide and dexamethasone. After an additional 3 months, the monoclonal protein became undetectable, with sustained remission of diarrhea after treatment discontinuation. The temporal concordance with monoclonal protein kinetics is shown in Figure 1A.

### Case 2

A 57-years-old man presented with a more than 2-years history of chronic diarrhea and hypoalbuminemia of unclear etiology. Over the preceding 2 months his symptoms worsened, with yellow watery stools occurring five to ten times daily, including nocturnal episodes, accompanied by intermittent abdominal distension and pain. He had lost approximately 10 kg, with a body mass index of 20.7 kg/m^2^. Previous investigations including repeated endoscopic and radiologic evaluations had not established a diagnosis. Stool studies excluded steatorrhea, and dietary elimination of lactose and gluten produced no improvement. Treatment with antibiotics and antidiarrheal medications was ineffective.

**FIGURE 2:**
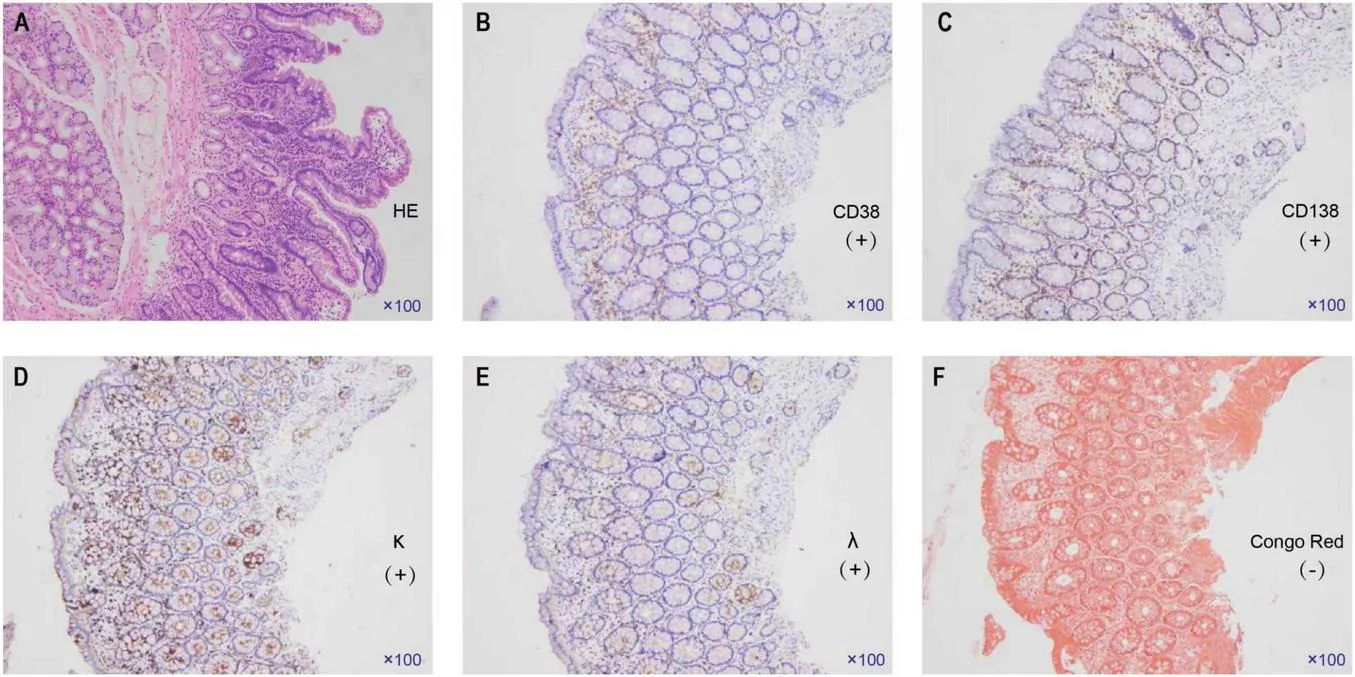
Representative small intestinal histopathology in Case 2. **(A)** Hematoxylin and eosin staining demonstrates preserved villous architecture without villous blunting and diffuse lymphoplasmacytic expansion of the lamina propria. **(B,C)** CD 38 and CD138 immunohistochemistry highlights increased plasma cells within the lamina propria. **(D,E)** Kappa and lambda light-chain staining shows balanced expression, consistent with a polyclonal plasma cell population. **(F)** Congo red staining is negative, excluding amyloid deposition.

Upper and lower endoscopy revealed chronic nonatrophic gastritis, mucosal abnormalities of the duodenal bulb, and multiple polypoid lesions in the colon and rectum. Histologic evaluation of intestinal biopsies demonstrated preserved villous architecture without villous blunting or increased intraepithelial lymphocytes. The lamina propria showed diffuse lymphoplasmacytic infiltration. Immunohistochemistry demonstrated abundant plasma cells (CD38^+^ and CD138^+^). Light-chain staining revealed balanced κ and λ expression, indicating a polyclonal plasma cell population. Congo red staining was negative for amyloid deposition (Figure 2). Immunostaining confirmed MUM1^+^ plasma cells with partial IgG expression and only scattered IgG4-positive plasma cells (IgG4/IgG < 10%). Hematologic evaluation identified an IgA-κ monoclonal protein (2.4 g/L). Bone marrow examination revealed 5.5%–8% mature plasma cells without evidence of clonality by immunohistochemistry or flow cytometry. The serum free light-chain ratio was mildly elevated (κ/λ ratio 2.08).

Given the presence of monoclonal gammopathy and the absence of an alternative explanation for the patient’s enteropathy, plasma cell–directed therapy with lenalidomide and dexamethasone was initiated. Clinical improvement occurred within 3 days, and diarrhea resolved completely within 1 week. Follow-up testing after 3 months demonstrated reduction of the monoclonal protein along with normalization of serum albumin (Figure 1B). A structured summary of the clinical, hematologic, and pathological features of both cases is provided in Table 1.

**TABLE 1:** Clinical, hematologic, and pathologic features of patients with monoclonal gammopathy associated enteropathy.

Feature	Case 1	Case 2
Demographics		
Age	63	57
sex	Male	Male
Clinical presentation		
Duration of diarrhea	3 years	2 years
Stool frequency (baseline)	4–8/day	5–10/day
Hypoalbuminemia	Yes	Yes
Response to dietary modification	No improvement with gluten- or dairy-free diet	No improvement with gluten- or dairy-free diet
Stool studies		
Occult blood	Negative	Negative
Fecal leukocytes	Absent	Absent
Fecal fat	Not increased	Not increased
Hematologic findings		
M-protein isotype	IgG κ	IgA κ
M-protein level (g/L)	8.9	1.4
Bone marrow plasma cells	2%	5.5%–8%
Gastrointestinal evaluation		
Endoscopic findings	Small bowel endoscopy revealed multiple ulcers	Chronic non-atrophic gastritis; polypoid lesions in the colon and rectum
Intestinal pathology		
Chronic inflammatory changes	Present	Present
Lamina propria plasma cell infiltration	Prominent	Prominent
Plasma cell clonality (κ/λ)	Polyclonal	Polyclonal
IgG4-positive plasma cells	Scattered	Scattered
Amyloid deposition (Congo red)	Negative	Negative
Treatment and response		
Plasma cell–directed therapy	TCd VCd	Rd
Time to diarrhea improvement	1 Week	3 Days
M-protein response	Decreased	Resolved
Histopathologic features		
Villous architecture	Preserved	Preserved
Intraepithelial lymphocytes	Not increased	Not increased
Lamina propria inflammation	Diffuse lymphocytes and plasma cells	Diffuse lymphocytes and plasma cells
Goblet and Paneth cells	Preserved	Preserved

## Discussion

Chronic inflammatory diarrhea with preserved intestinal architecture remains diagnostically challenging when common causes, including inflammatory bowel disease, infection, and autoimmune enteropathy, have been excluded. In the two patients presented here, extensive clinical, endoscopic, histopathologic, and laboratory investigations failed to identify an established cause of enteropathy. Both patients demonstrated a similar intestinal phenotype characterized by preserved villous architecture, absence of crypt distortion or epithelial apoptosis, and diffuse lymphoplasmacytic inflammation enriched with polyclonal plasma cells. Importantly, both patients had a circulating monoclonal immunoglobulin and experienced rapid clinical improvement after plasma cell–directed therapy, accompanied by reduction of the monoclonal protein.

The diagnosis of monoclonal gammopathy associated enteropathy requires careful exclusion of alternative causes. In these cases, inflammatory bowel disease was considered unlikely because of the absence of chronic architectural distortion, crypt destruction, or neutrophil-predominant inflammation. Autoimmune enteropathy was considered less likely given the preserved epithelial integrity and absence of significant crypt apoptosis. Infectious causes were excluded through comprehensive microbiological evaluation. Other immune mediated and malabsorptive disorders were also investigated. Histologic findings did not support microscopic colitis, as there was no characteristic increase in intraepithelial lymphocytes, subepithelial collagen deposition. Seronegative celiac disease was considered unlikely because of preserved villous architecture and absence of increased intraepithelial lymphocytes. There was no clinical or laboratory evidence supporting common variable immunodeficiency–associated enteropathy, and medication-related enteropathy was excluded based on medication history and lack of response to withdrawal or conventional supportive treatment. Together, these features delineate a distinct clinicopathologic phenotype not encompassed by current classifications of inflammatory enteropathies.

In 2025, we reported a patient with long-standing chronic diarrhea attributed to monoclonal gammopathy, which was described at that time as an atypical manifestation within the spectrum of monoclonal gammopathy of clinical significance (MGCS) (3).

A major challenge in interpreting these cases is determining whether the monoclonal gammopathy represents a pathogenic driver or an incidental finding. Monoclonal gammopathies are common in older individuals, and coexistence alone does not establish causality. However, increasing evidence from the spectrum of monoclonal gammopathy of clinical significance (MGCS) indicates that monoclonal immunoglobulins may contribute to organ dysfunction through mechanisms beyond direct tissue deposition or clonal infiltration. Examples include complement-mediated injury, autoantibody activity, and immune dysregulation (4, 5). Therefore, the absence of intestinal monoclonal plasma cell infiltration or immunoglobulin deposition does not exclude a pathogenic role of the circulating monoclonal protein.

The rationale for plasma cell directed therapy was based on the coexistence of monoclonal gammopathy, severe treatment refractory diarrhea without an alternative explanation, and the possibility that the monoclonal protein contributed to intestinal inflammation. The parallel improvement in symptoms and reduction of monoclonal protein after therapy support an association, although causality cannot be established. Nonspecific immunomodulatory effects of treatment cannot be excluded, and functional studies are needed to define the pathogenic role of monoclonal immunoglobulins.

These findings suggest that monoclonal gammopathy associated enteropathy may represent a potential manifestation within the spectrum of MGCS. Whether this phenotype represents a distinct entity or a heterogeneous subgroup remains to be determined in larger cohorts. Clinically, monoclonal gammopathy should be considered in patients with unexplained, treatment-refractory inflammatory diarrhea and plasma cell–rich intestinal inflammation lacking features of conventional enteropathies. Both patients achieved sustained remission after therapy (Case 1: 12 months; Case 2: 3 months), although longer follow-up and independent validation are required to define the durability and clinical significance of this association.

## Data Availability

The original contributions presented in the study are included in the article, further inquiries can be directed to the corresponding author.
